# The Dose–Response Association between Nitrogen Dioxide Exposure and Serum Interleukin-6 Concentrations

**DOI:** 10.3390/ijms18051015

**Published:** 2017-05-08

**Authors:** Jennifer L. Perret, Gayan Bowatte, Caroline J. Lodge, Luke D. Knibbs, Lyle C. Gurrin, Rangi Kandane-Rathnayake, David P. Johns, Adrian J. Lowe, John A. Burgess, Bruce R. Thompson, Paul S. Thomas, Richard Wood-Baker, Stephen Morrison, Graham G. Giles, Guy Marks, James Markos, Mimi L. K. Tang, Michael J. Abramson, E. Haydn Walters, Melanie C. Matheson, Shyamali C. Dharmage

**Affiliations:** 1 Allergy and Lung Health Unit, Center for Epidemiology and Biostatistics, the University of Melbourne, Melbourne, Victoria 3010, Australia; gayan.bowatte@unimelb.edu.au (G.B.); clodge@unimelb.edu.au (C.J.L.); lgurrin@unimelb.edu.au (L.C.G.); lowe.adrian@gmail.com (A.J.L.); jburgess@unimelb.edu.au (J.A.B.); Haydn.Walters@utas.edu.au (E.H.W.); mcmat@unimelb.edu.au (M.C.M.); s.dharmage@unimelb.edu.au (S.C.D.); 2 Institute for Breathing and Sleep (IBAS), Heidelberg, Melbourne, Victoria 3084, Australia; 3 School of Public Health, the University of Queensland, Herston, Queensland 4006, Australia; l.knibbs@uq.edu.au; 4 School of Clinical Sciences at Monash Health, Monash University, Melbourne, Victoria 3004, Australia; rangi.kandane-rathnayake@monash.edu; 5 School of Medicine, University of Tasmania, Hobart, Tasmania 7001, Australia; david.johns@utas.edu.au (D.P.J.); Richard.WoodBaker@utas.edu.au (R.W.-B.); 6 “Breathe Well” Center of Research Excellence for Chronic Respiratory Disease and Lung Ageing, School of Medicine, University of Tasmania, Hobart, Tasmania 7005, Australia; 7 Allergy, Immunology and Respiratory Medicine, the Alfred Hospital, Melbourne, Victoria 3004, Australia; B.Thompson@alfred.org.au; 8 Prince of Wales’ Hospital Clinical School and School of Medicine Sciences, Faculty of Medicine, University of New South Wales, Sydney, NSW 2052, Australia; paul.thomas@unsw.edu.au; 9 Department of Medicine, University of Queensland, Brisbane, Queensland 4072, Australia; stephen.morrison@uq.edu.au; 10 Cancer Epidemiological Center, Cancer Council Victoria, Melbourne, Victoria 3053, Australia; graham.giles@cancervic.org.au; 11 South West Sydney Clinical School, the University of NSW, Liverpool, NSW 2170, Australia; guy.marks@sydney.edu.au; 12 Department of Respiratory Medicine, Launceston General Hospital, Launceston, Tasmania 7250, Australia; jamesmarkos@bigpond.com; 13 Department of Allergy and Immunology, Royal Children’s Hospital, Parkville, Victoria 3052, Australia; mimi.tang@mcri.edu.au; 14 Allergy and Immune Disorders, Murdoch Children’s Research Institute, Parkville, Victoria 3052, Australia; 15 Department of Paediatrics, the University of Melbourne, Victoria 3010, Australia; 16 School of Public Health & Preventive Medicine, Monash University, Melbourne, Victoria 3004, Australia; Michael.Abramson@monash.edu

**Keywords:** nitrogen dioxide, traffic-related air pollution, interleukin, tumor necrosis factor-α, systemic inflammation, airflow obstruction

## Abstract

Systemic inflammation is an integral part of chronic obstructive pulmonary disease (COPD), and air pollution is associated with cardiorespiratory mortality, yet the interrelationships are not fully defined. We examined associations between nitrogen dioxide (NO_2_) exposure (as a marker of traffic-related air pollution) and pro-inflammatory cytokines, and investigated effect modification and mediation by post-bronchodilator airflow obstruction (post-BD-AO) and cardiovascular risk. Data from middle-aged participants in the Tasmanian Longitudinal Health Study (TAHS, *n* = 1389) were analyzed by multivariable logistic regression, using serum interleukin (IL)-6, IL-8 and tumor necrosis factor-α (TNF-α) as the outcome. Mean annual NO_2_ exposure was estimated at residential addresses using a validated satellite-based land-use regression model. Post-BD-AO was defined by post-BD forced expiratory ratio (FEV_1_/FVC) < lower limit of normal, and cardiovascular risk by a history of either cerebrovascular or ischaemic heart disease. We found a positive association with increasing serum IL-6 concentration (geometric mean 1.20 (95% CI: 1.1 to 1.3, *p* = 0.001) per quartile increase in NO_2_). This was predominantly a direct relationship, with little evidence for either effect modification or mediation via post-BD-AO, or for the small subgroup who reported cardiovascular events. However, there was some evidence consistent with serum IL-6 being on the causal pathway between NO_2_ and cardiovascular risk. These findings raise the possibility that the interplay between air pollution and systemic inflammation may differ between post-BD airflow obstruction and cardiovascular diseases.

## 1. Introduction

In 2012, the World Health Organization (WHO) attributed exposure to outdoor air pollution as the cause for 389,000 premature deaths related to chronic obstructive pulmonary disease (COPD) [1]. The corresponding air pollution-related estimate that related to ischaemic heart disease and stroke was five-fold higher, contributing to 72% of the 3 million premature deaths worldwide [2]. A recent meta-analysis of 13 studies from across North America, Europe and Asia has shown a modest increase in the relative risk (RR) of respiratory mortality with increasing chronic exposure to nitrogen dioxide (NO_2_) (RR 1.02 (95% confidence interval (CI): 1.02–1.03) per 10 µg/m^3^ (or equivalent to per 5.32 ppb increase)). This estimate comparable to the major air pollutant, particulate matter less than 2.5 µm in diameter (PM_2.5_) (RR 1.05 (1.01–1.09) per 10 µg/m^3^) [3]. In this same meta-analysis, the positive association between NO_2_ concentration and cardiovascular (CV) mortality was significantly stronger than for respiratory mortality, in spite of marked heterogeneity between studies [3].

Systemic oxidative stress and inflammation have been implicated as a potential biological pathway of pollution-related cardiovascular effects. This process originates in the lung and especially involves the pro-inflammatory cytokines, interleukin (IL)-6 and tumor necrosis factor-α (TNF-α) [4]. Epidemiological research has found only moderate evidence for a systemic inflammatory response to traffic-related air pollution (TRAP) given a predominance of studies that addressed short-term exposures to PM_2.5_ [5]. As a gaseous air pollutant, nitrogen dioxide (NO_2_) is regarded as a strong marker for air pollution primarily generated from combustion including motor vehicles, biomass burning, airports and industry [6,7]. Associations with NO_2_ exposure were not reported by the Harvard Six Cities Study, a major longitudinal population-based study that has provided strong evidence to support a causal relationship between PM_2.5_ and cardiovascular risk [5,8,9]. The epidemiological data regarding the relationship between NO_2_ exposure and chronic obstructive pulmonary disease (COPD), an inflammatory lung condition that is characterized by progressive airflow obstruction (AO), are limited [10].

Of the endogenous pro-inflammatory cytokines, IL-6 is a key player, particularly as the primary inducer of C-reactive protein (CRP). Although neither are routinely measured as part of risk prediction for coronary heart disease (CHD) [11], CRP (and IL-6 in unadjusted models) are associated with an increased CHD risk [12]. IL-6 regulates many pathways involved in the acute phase response and adaptive immunity [13], and can be a marker of COPD progression [14,15,16], especially when persistently elevated in people with otherwise stable disease [15]. A recent meta-analysis of 33 individual studies found increased serum IL-6 among people with COPD compared with controls [17]; however, there was evidence of publication bias and no account for diurnal variation [18] or smoking status [19]. While TRAP might have direct effects on cardiorespiratory disease and IL-6 separately, it might also be possible that CV disease and/or COPD influence IL-6 levels following TRAP exposure as an indirect effect. In other words, CV disease and/or COPD may in part mediate an increase in serum IL-6 in response to higher NO_2_ exposures and which, in turn, might adversely influence subsequent cardiorespiratory outcomes. In addition to IL-6, the roles of other classical pro-inflammatory cytokines such as IL-8 and TNF-α in these interactions are unknown, although notably, COPD patients have been shown to have lower levels of these cytokines than smokers without COPD [15].

Using data from middle aged participants of the fifth decade follow-up of the Tasmanian Longitudinal Health Study (TAHS), we aimed to clarify interrelationships between NO_2_, systemic cytokines and specific cardiorespiratory condition by means of: (A) examining cross-sectional relationships between NO_2_ exposure and the pro-inflammatory cytokines, IL-6, IL-8 and TNF-α; and (B) examining for effect modification and/or mediation by post-bronchodilator (BD)-AO and cardiovascular disease.

## 2. Results

### 2.1. Characteristics of TAHS Participants

Clinical characteristics and lung function data for the 1389 laboratory study participants have been published previously [20]. Briefly, the mean age (standard deviation (SD)) was 44.9 [0.85] years and 51% were male. This group was enriched for asthma-ever, wheezy breathing and/or chronic bronchitis, with one-quarter reporting asthma symptoms or medication use within the last 12 months (*n* = 335). Over half were ever-smokers (57%, *n* = 804), of whom 59% (*n* = 457) had smoked for at least ten pack-years. The criterion for post-BD-AO was met by 9.25% (*n* = 123), for whom mild, moderate and severe airflow obstruction was identified for 77% (*n* = 95), 10% (*n* = 12) and 13% (*n* = 16) of participants, respectively.

Of these participants with post-BD-AO of any severity, 87 (71%) reported cough, wheeze and/or sputum expectoration; 71 (58%) had a 10-pack-year smoking history; and 66 (55%) had either asthma symptoms or had taken asthma medication within the last 12 months. Of the 828 participants of the subsequent laboratory visit, only 2.2% (19/828) reported a history of CVD, of whom 12 (63%) reported ischaemic heart disease and seven (37%) reported cerebrovascular disease.

Descriptive statistics for participants with complete data have been summarized in Table 1, stratified by the presence or absence of post-BD-AO. Expected differences in ventilatory function and cumulative smoking history were seen; otherwise, variations in annual mean NO_2_ and measured cytokine concentrations were not statistically significant.

### 2.2. Main Association between NO_2_ Exposure and Cytokines

For all participants, increasing annual mean NO_2_ exposure when expressed as quartiles was associated with increasing concentrations of serum IL-6 (Table 2, *p*-for-univariable trend = 0.012). Compared with participants exposed to the lowest quartile of annual mean NO_2_, those exposed to the second highest and highest quartiles had higher serum IL-6 concentrations with the ratio of geometric mean (GM) estimated at 1.46 and 1.71, respectively. When quartiles of NO_2_ exposure were expressed continuously, the estimate for serum IL-6 per quartile increase in annual mean NO_2_ concentration was GM 1.20 (95% CI: 1.1 to 1.3, *p* = 0.001).

However, main effects were not seen for either IL-8 or TNF-α (*p*-for-univariable trend = 0.852 and 0.710, respectively). When quartiles of NO_2_ exposure were expressed continuously, the adjusted estimate per quartile increase in annual mean NO_2_ concentration was GM 1.06 ((95% CI: 0.95, 1.17), *p* = 0.297), for serum IL-8, and GM 1.04 ((0.99, 1.1), *p* = 0.13) for TNF-α.

No interactions were seen between the effects of NO_2_ exposure and post-BD-AO on any of the circulating pro-inflammatory cytokines (*p*-for-interaction > 0.174). There were no NO_2_-CVD interactions (*p*-for-interaction > 0.178); however, only a few participants reported ischaemic cardiovascular events in this age group (*n* = 19).

### 2.3. Mediation Analysis by Post-BD-AO

We used Causal Mediation Analysis [21] to determine the extent to which the total effect of NO_2_ exposure on the pro-inflammatory cytokines was mediated by post-BD-AO, as measured by a continuous variable. This analysis partitioned the total effect into an indirect effect of NO_2_ on cytokines (NO_2_ acting through post-BD-AO and then post-BD-AO on cytokines) and a direct effect of NO_2_ on cytokines (that did not act through changes in post-BD-AO induced by changes in NO_2_ exposure).

We observed only a direct relationship between NO_2_ exposure and serum IL-6 concentration, and a negligible indirect effect through post-BD-AO (Table 3). The estimate suggested that around 17.5% of the variation in IL-6 was explained by a direct effect from NO_2_ exposure. As there was little evidence of a univariate relationship between serum IL-6 and post-BD-AO when lung function was analysed as the outcome (*p* = 0.145), this suggested that it was unlikely that IL-6 was on the causal pathway between NO_2_ exposure and post-BD-AO.

In the absence of main effects between NO_2_ exposure and serum IL-8 and TNF-α, the mediation effects on these cytokines were effectively negligible.

### 2.4. Mediation Analysis by Cardiovascular Disease (CVD)

**Figure A1:**
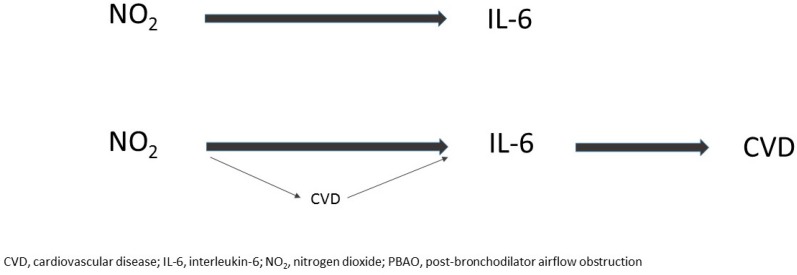
Potential representation of NO_2_ exposure, systemic IL-6 concentrations and its relationships with post-BD airflow obstruction and cardiovascular disease.

We observed a modest direct relationship between NO_2_ exposure and serum IL-6 concentrations (10.8% (95% CI: −6 to +26)), with a correspondingly minor indirect effect through CVD (0.6% (−1 to +2.8)). In other words, of the total effect of NO_2_ exposure on serum IL-6 (11.4% (−7 to +29)), the proportion of the total effect mediated was 0.054 or 5.4%. In separate univariate models when CVD was analysed as the outcome, we additionally saw both serum IL-6 (OR 2.24 (95% CI: 1.2, 4.1, *p* = 0.008) and IL-8 concentrations (OR 2.05 (1.1, 3.9), *p* = 0.029) associated with cardiovascular risk. However, in the context of the main association between NO_2_ and serum IL-6 concentrations (Table 2), these observations are consistent with circulating IL-6 being on the causal pathway between NO_2_ exposure and cardiovascular risk (summarised in Figure A1).

## 3. Discussion

Our cross-sectional analysis of the fifth decade follow-up of the TAHS longitudinal cohort has shown a dose–response relationship between NO_2_ exposure and serum IL-6 levels at relatively low levels of outdoor air pollution that was not evident for either IL-8 or TNF-α. However, there was little evidence of either a modifying or mediating effect for the lung function criterion for diagnosing COPD, post-BD-AO. Furthermore, the lack of association between IL-6 and post-BD-AO as the outcome effectively excludes IL-6 being on the causal pathway. In contrast, our findings raise the possibility that circulating IL-6 might be on the causal pathway between NO_2_ exposure and cardiovascular risk.

Activation of oxidative stress and the inflammatory response in both the pulmonary and systemic compartments is one of the biological pathways postulated to mediate the adverse cardiovascular effects of outdoor air pollution [4,5]. However, not all epidemiological data support this premise [22]. Our finding of a dose–response main association between NO_2_ exposure and serum IL-6 levels is consistent with the linear relationship that has been seen for PM_2.5_ as adverse health effects occur at low levels of exposure [8,9]. Although this relationship starts to become less steep at very high concentrations of air pollutants, it becomes steeper at lower concentrations [23]. The lack of a currently recognised threshold value reflects the 2005 WHO guidelines that aimed to limit particulate matter concentrations to the lowest possible level [2].

Australia has relatively low levels of combustion-derived air pollution with the national population-weighted average concentrations of NO_2_ ranging between 7.3 ppb (2006) and 6.3 ppb (2011) [6]. In the present study, the positive association between NO_2_ concentration and serum IL-6 plus the observation of IL-6 acting on cardiovascular risk favors a predisposition to cardiovascular events for residents of more polluted areas [3,24]. In contrast, the relative lack of an effect mediated through post-BD-AO (and cardiovascular risk) supports a predominant direct effect of NO_2_ on circulating IL-6 levels, although a high proportion of the IL-6 variation is likely to represent unexplained factors. While potential biological pathways with systemic IL-6 have been somewhat clarified using mediation analysis, it is insufficient to explain the NO_2_-related increase in cardiorespiratory mortality [3,25].

For COPD patients, systemic inflammation has been identified to be an integral component of the phenotype associated with accelerated lung function decline [26] and increased all-cause mortality [15]. However, the interplay between outdoor air pollution, post-BD airflow obstruction and inflammatory processes has not been well characterized [27]. In a study of 242 clinically stable COPD patients, NO_2_ exposure was associated with elevated levels of CRP, but not its inducer IL-6 [10], although it was not clear whether the blood sampling in the early morning hours spanned a period when IL-6 levels might be quite variable as exact times were not given [18].

Whilst TRAP might indirectly influence systemic inflammation responses through a predisposition to infection [28,29], our findings are consistent with a positive and direct association between NO_2_ exposure and systemic IL-6 concentrations at a population level. This relationship was not augmented for the subgroup who fulfilled the lung function criterion for the clinical COPD phenotype, although the majority of participants with post-BD-AO in the present study only had mild-to-moderate disease. The lack of main effects for serum IL-8 and TNF-α may reflect the lower persistent levels seen in people with COPD when compared with smokers who do not have the disease [15].

Our study has three main strengths: first, our measure of TRAP was derived from a validated satellite-based land-use regression model for NO_2_ with national coverage [6], as ground-based monitoring networks typically have sparse spatial coverage [30]. Second, our study was based on prospectively collected data from a large population-based sample with a relatively low attrition (around 33%) between 1968 (*n* = 8583) and 2004 (*n* = 5729), so estimates may represent the general population after adjusting for sampling weights and smoking history. Third, although blood sampling is typically taken in the early morning after fasting overnight [15] to minimize the diurnal variation, the present study neither controlled for fasting nor for time of blood sampling. We still were able to standardize cytokine concentrations based on the known diurnal variation of IL-6 in the regression models [18], although this might have introduced some measurement bias.

Our study also had limitations: firstly, the cross-sectional analysis has assessed for relevant associations, but the lack of temporality does not support causal inference. We were unable to assess accelerated FEV_1_ decline as an outcome, which is a key feature of the clinical COPD phenotype. In addition, our annual NO_2_ exposure variable would have been strengthened if the measurement we used encompassed a longer period of exposure, and if seasonal NO_2_ variation (in addition to season of blood sampling) was taken into account [31]. Secondly, although we made some adjustment for time to processing, and followed methods for optimizing serum storage duration and conditions [32,33], it is probable that absolute cytokine concentrations were affected by a degree of degradation, absorption and/or cellular production [34], especially with regard to TNF-α which has a half-life of only hours [35]. Thirdly, due to the relatively young age of participants, there were low case numbers in the group reporting cardiovascular events which most likely resulted in insufficient power to examine for effect modification of the NO_2_-IL-6 relationship. Fourthly, peripheral blood mononuclear cells (PBMCs) were not isolated at the time of blood collection, and so additional information was not obtained to more fully describe related immunological processes. Finally, as the laboratory participants resided in urban areas of Australia and were almost exclusively Caucasian, this might limit the generalizability of our findings to other populations.

## 4. Materials and Methods

### 4.1. Study Design and Population

**Figure 1:**
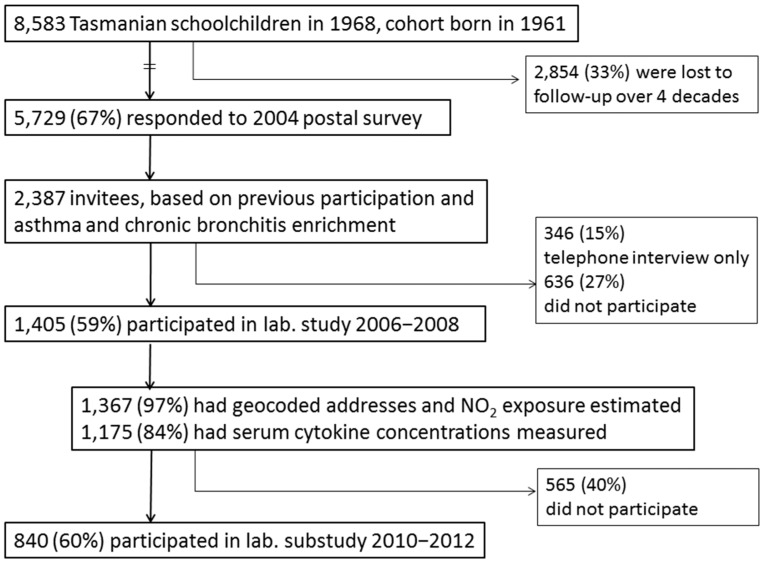
Tasmanian Longitudinal Health Study flow diagram.

The subjects were participants in the 5th decade follow-up of the population-based TAHS, which commenced in 1968. The methods for the baseline and subsequent follow-up studies have been published [20,32,36,37,38,39,40]. Briefly, the participants (otherwise known as “probands”) who were born in 1961 and schooled in Tasmania in 1968 (*n* = 8583 (99%)) were studied by questionnaires and spirometry when seven-years old. Of the 7312 (85%) original probands who were retraced between 2002 and 2004, a detailed postal survey was then conducted when the probands were aged in their early-to-mid forties. A total of 5729 (78%) responded, representing two-thirds (67%) of the original cohort. Respondents were chosen to participate in a clinical laboratory study based on either participation in multiple follow-up studies (including the 1974 clinical study and/or 1992 follow-up visits), or on the presence of mid-adult asthma and/or chronic bronchitis. Of the 2373 invited, 1389 (58.5%) participated by completing a detailed questionnaire and undergoing blood sampling as well as complex lung function and skin prick testing. The main analysis here was based on the questionnaire data collected in 2004–2008, and the measurement of post-bronchodilator (BD) spirometry for those who participated in the clinical study between 2006 and 2008 (*n* = 1389). For a subset of these participants (*n* = 837), additional data on history of cardiovascular events was obtained from the 2010 clinical study (see definition of cardiovascular risk). These main components of the study design have been illustrated in Figure 1 and in reference [39], Figure 1 on page 4.

### 4.2. Data Collection Methods

Details of lung function testing have been described elsewhere [20]. The measurements of lung function were standardized across testing sites following American Thoracic Society (ATS) and European Respiratory Society (ERS) standards [41]. The predicted values for spirometry were calculated using reference values from the Global Lung function Initiative [42].

Residential addresses of participants who attended the laboratory study were geocoded for 1367 (98.4%) using the Geocoded National Address File (GNAF) [43]. Mean annual ambient (i.e., outdoor) nitrogen dioxide (NO_2_) concentrations were estimated using a validated satellite-based land-use regression (LUR) model [6]. The LUR model utilized satellite observations of tropospheric NO_2_ columns combined with ground-based predictors of NO_2_, such as land use and roads, to estimate ground-level NO_2_ across Australia. This model was able to capture 81% of the spatial variation in annual NO_2_ levels with a cross-validation prediction error of 19% for the study period. This measure of NO_2_ was used for all analyses. The annual mean concentration of NO_2_ corresponded to the year of lung function testing and serum cytokine measurement.

### 4.3. Clinical Definitions

Personal smoking in 2004 was defined by the question “In your lifetime, have you smoked at least 100 cigarettes or equal amounts of cigars, pipes or other tobacco product?”, and was expressed by smoking status (never, past, current), and smoking duration and intensity (pack-years). One pack-year was equivalent to smoking 20 cigarettes per day for one year. Pack-years were categorized into none, 0 < pack-years < 15, 15 ≤ pack-years < 30 and 30 + pack-years.

Post-bronchodilator airflow obstruction (Post-BD-AO) was defined by FEV_1_/FVC less than the fifth percentile of the normal predicted values (*z*-score < −1.645 standard deviations) following 200 μg of salbutamol administered via spacer, and categorized according to severity [44].

Cardiovascular risk was defined by an affirmative response to the question/s from the clinical follow-up in 2010: (1) “Has a doctor ever told you that you have/had angina, heart attack or myocardial infarction?” or (2) “Has a doctor ever told you that you have/had a transient ischaemic attack (TIA) or a stroke?”

Body mass index (kg/m^2^) was calculated, and using World Health Organization criteria, categorized into underweight (<18.5), normal (18.5 to <25), overweight (25 to <30) and obese (30+) subgroups.

### 4.4. Cytokine Measurement

The laboratory methods have been described in detail by Kandane-Rathnayake and colleagues [45]. In brief, whole blood was collected in serum tubes, spun at 2500 rpm for 15 min and serum was stored at −80 °C until the vial was thawed for testing. The average time from blood collection to processing and freezing was 1.8 days, and the blood collection times ranged between 08:30 and 20:30 h with a peak between 18:45 and 19:00 h. Serum levels of cytokines were measured using a high sensitivity Lincoplex assays (LINCO Research, St. Charles, MI, USA) and Luminex Technology (Luminex Corporation, Austin, TX, USA).

The 96-well microtitre filter plates were coated with microspheres and incubated overnight with 50 μL of standards, controls and blood serum samples. The plates were incubated with detection antibody, which comprised a mixture of biotinylated mouse/rat anti-human cytokine antibodies directed against each cytokine, and this was followed by streptavidin-conjugated phycoerythrin (PE). The microspheres were resuspended in sheath fluid and the fluorescence output was read on the Luminex instrument (Luminex Corporation, Austin, TX, USA) in eight batches.

Samples with readings below the minimum detection limit for each assay at 0.13 pg/mL were assigned a value of half this value (0.065 pg/mL), whereas samples with readings at or above the maximum value of 2000 pg/mL were assigned this value (2000 pg/mL). This was relevant to 5.9% and 0.3% of samples for IL-6, respectively, 0% and 3.5% for IL-8, respectively, and 0.4% and 0% for TNF-α, respectively, so a ceiling effect was relevant for IL-8. Samples with undetectable readings were evenly distributed between batches, and sensitivity analyses did not significantly change the results [45].

Most analyses were performed singly, and this decision was based on the results from a subset that was measured in duplicate (*n* = 79). The reliability was determined by the Intraclass Correlation Coefficient, which was above 97%, except for TNF-α (IL-6, 98.6%; IL-8, 97.7%; TNF-α, 92.7%). Two quality controls were used for each cytokine and run in duplicate in each batch, and all were within the expected range as coefficients of variation were less than 15%.

### 4.5. Statistical Analysis

All analyses were carried out using Stata (release 14SE, Stata Corporation, College Station, TX, USA). Patient characteristics, NO_2_ concentrations, post-BD spirometry and serum cytokine concentrations were compared between patients with and without post-BD-AO using chi-squared tests for categorical variables and Wilcoxon rank sum tests for continuous variables. We examined the association between NO_2_ and serum cytokines concentrations using linear regression models that incorporated the bootstrap method to estimate robust standard errors. Cytokine concentrations were positively skewed; therefore, values were log_10_ transformed in order to perform linear regression, and results were reported as geometric mean with corresponding 95% confidence intervals. For the cross-sectional analyses examining main effects, NO_2_ concentration was expressed in quartiles and analyzed as a continuous variable if the assumption of linearity was fulfilled. For the interaction and mediation analyses, NO_2_ exposure was primarily expressed as a binary variable by determined by a cut-off between the highest and second-highest quartile (0 < NO_2_ < 5.8 and > 5.8 ppb), which is roughly comparable to the 10 µg/m^3^ concentration used by previous meta-analyses [3,24]. Those with missing data were excluded from individual analyses, consistent with complete case analysis.

Multivariable regression models were adjusted a priori for sex, pack-year history and smoking status, and highest attained levels of education. Sampling weights were used a priori given the asthma and chronic bronchitis-enrichment, where sampling weights were the inverse of the probability of being included in the sample, defined as the number in the strata divided by the number of selected from each stratum. Body mass index was considered to be a potential confounder and was included in models if the estimate changed by ≥10%. To standardize the variability of the serum cytokine measurements, the season of study attendance and batch number were also included a priori. The diurnal variation of IL-6 was addressed by representing Maggio and colleagues [18] with the following approximate times of blood collection over the course of the day: 08:30–10:00 h (including a collection at 07:55 h); 10:01–12:00 h; 12:01–13:00 h; 13:01–14:00 h; 14:01–15:00 h, 15:01–16:30 h and 16:31–20:00 h. As circulating IL-8, but not TNF-α, is also known to have diurnal variation [46], this variable of “blood collection time” was included in the IL-8 analyses.

As potential biologically plausible effect modifiers, two-way interactions between the effects of NO_2_ exposure and post-BD-AO, and the effects of NO_2_ and cardiovascular history were assessed with regard to serum cytokine concentrations. Causal mediation analysis was performed using the medeff command in Stata to assess how much of the presumed causal association between NO_2_ exposure and the pro-inflammatory cytokines was mediated by post-BD-AO and/or cardiovascular history [47]. A conventional cut-off of *p* < 0.05 was used to determine statistical significance for all analyses.

### 4.6. Ethics

This study was approved by the Human Ethics Review Committees at The Universities of Melbourne (approval number 040375), Tasmania (040375.1) and New South Wales (08094), the Alfred Hospital (1118/04), and Royal Brisbane and Women’s Hospital Health Service District (2006/037). Written informed consent was obtained from all participants. No project identification code has been linked to this study as the TAHS originated in 1968.

### 4.7. Data Sharing

The TAHS is a cohort study with data that has been prospectively collected since 1968, and will be an ongoing resource for future epidemiological analyses. Data collection and protocols have been detailed in the TAHS cohort profile paper published in 2016 [39]. The raw data have not been made widely available here, but expressions of interest can be discussed with the corresponding author, Jennifer L. Perret and/or the principal investigator, Shyamali C. Dharmage, on an individual basis.

## 5. Conclusions

In a cross-sectional analysis of middle-aged adults, we have described an incremental pattern of pollution-related responses for serum IL-6 with regard to NO_2_ exposure, which was not observed for other pro-inflammatory cytokines, namely, IL-8 and TNF-α. Although case numbers were limited, we have shown that this NO_2_-IL-6 relationship was neither modified nor mediated by the presence of post-BD-AO. In contrast to this lack of influence from post-BD-AO, serum IL-6 was found to act on cardiovascular risk, which is consistent with IL-6 being on the causal pathway between NO_2_ and cardiovascular disease. While it is important to examine the cardiorespiratory effects of pollutant co-exposures including PM_2.5_, overall, these findings reinforce public health recommendations to reduce exposure to outdoor air pollutants. This includes the avoidance of combustion-derived pollution from gasoline and diesel, industry and sources of biomass burning, which is relevant even in low-pollution settings.

## Figures and Tables

**Table 1:** Characteristics of the TAHS laboratory study participants, by post-bronchodilator airflow obstruction.

Participant Characteristic	TAHS 5th Decade Laboratory Study		
	Post-BD Airflow Obstruction (*n* = 973) ^1^		
	Yes (*n* = 97)	No (*n* = 876)	*p*-Value
Demographic and other features			
Age in years (mean (SD))	44.9 (0.8)	44.9 (0.9)	0.153
Sex (*n*, % male)	50 (52)	438 (50)	0.773
BMI categories (kg/m^2^)			
Underweight (<18.5)	1 (1)	3 (0.3)	0.315
Normal (18.5–24.9)	30 (31)	262 (30)	0.835
Overweight (25–29.9)	36 (37)	359 (41)	0.462
Obese (≥30)	30 (31)	252 (29)	0.498
Cigarette smoking			
None	21 (22)	389 (45)	<0.001
Past, <20 pack years	16 (17)	211 (24)	0.107
Past, ≥20 pack years	4 (4)	47 (5)	0.624
Current, <20 pack years	17 (18)	115 (13)	0.206
Current, ≥20 pack years	37 (39)	109 (13)	<0.001
Annual mean NO_2_ exposure			
Predicted ppb (median, IQR)	4.1 (3.5, 5.9)	4.2 (3.5, 5.6)	0.733
Per quartile (median, IQR)			
1 (lowest)	3.1 (2.8, 3.5)	3.1 (2.8, 3.4)	0.727
2	3.8 (3.7, 4.1)	3.9 (3.7, 4.1)	0.668
3	4.7 (4.6, 5.1)	4.9 (4.6, 5.4)	0.350
4 (highest)	6.7 (6.1, 7.3)	7.1 (6.3, 9.4)	0.066
Post-BD spirometry ^2^			
FEV_1_ (L)	2.84 (0.7)	3.47 (0.7)	<0.001
*z*-score	−1.44 (1.1)	0.015 (1.0)	<0.001
FVC (L)	4.45 (1.1)	4.34 (0.9)	0.542
*z*-score	+0.152 (1.1)	0.008 (0.9)	0.258
FEV_1_/FVC (ratio)	63.8 (7)	80.2 (4.7)	<0.001
*z*-score	−2.37 (0.7)	−0.041 (0.8)	<0.001
Serum cytokine concentrations [GM 95% CI] pg/mL ^†^			
Interleukin-6	13.9 (12.2, 15.7)	12.1 (8.2, 17.9)	0.795
Interleukin-8	275 (244, 308)	353 (245, 509)	0.861
Tumor necrosis factor-α	6.8 (6.5, 7.2)	6.1 (5.2, 7.3)	0.082

Definition of abbreviations: BD, bronchodilator; BMI, body mass index; CI, confidence interval; FEV_1_, forced expiratory volume in 1 s; FVC, forced vital capacity; GM, geometric mean; IQR, interquartile range; pg/mL, picograms per milliliter; ppb, parts per billion; NO_2_, nitrogen dioxide; TAHS, Tasmanian Longitudinal Health Study. ^1^ Participant numbers by complete case analysis with the following exclusions: NO_2_ not available (*n* = 64); no biospecimen (*n* = 214) or blood collection time recorded (*n* = 97); pack-years not recorded (*n* = 28); technically unacceptable spirometry (*n* = 60). ^2^ a *z*-score is the deviation from the mean predicted value expressed as standard deviations (SD), where 95% of normally distributed data lies between −1.96 SD and +1.96 SD.

**Table 2:** Dose–response relationship between annual mean NO_2_ exposure and serum interleukin-6 concentrations for all participants.

Annual Mean NO_2_ Exposure			Serum Interleukin-6 (95% CI)		*p*-Value
Quartile	Range (ppb)	*n*	GM pg/mL	Ratio of GM ^1^	
1	2.41–3.54	340	11.4 (9.0 to 14.5)	1	
2	3.54–4.30	340	12.9 (10.1 to 16.4)	1.16 (0.1 to 1.6)	0.400
3	4.31–5.81	338	16.0 (12.7 to 20.0)	1.46 (1.2 to 2.0)	0.024
4	5.81–23.8	337	17.5 (13.4 to 23.0)	1.71 (2.0 to 2.4)	0.003

Definitions of abbreviations: GM, geometric mean; NO_2_, nitrogen dioxide; ppb, parts per billion. ^1^ Multivariable models were adjusted for body mass index, smoking status, pack-year group, sex, highest education, sampling weights, blood collection time and delivery, seasons, batch (regression *n* = 973).

**Table 3:** Adjusted analyses for the NO_2_-cytokine relationship mediated by post-bronchodilator airflow obstruction ^1,2^.

Mediation Analysis Effect	Post-BD Airflow Obstruction as the Mediator between NO_2_ and Serum Pro-Inflammatory Cytokines (Mean (95% CI))		
	IL-6	IL-8	TNF-α
Indirect Effect	0.0003 (−0.007, 0.008)	0.003 (−0.02, 0.01)	−0.001 (−0.004, 0.01)
Direct Effect	0.175 (0.04, 0.30)	0.090 (−0.03, 0.20)	0.056 (0.003, 0.11)
Total Effect	0.175 (0.04, 0.30)	0.092 (−0.04, 0.20)	0.055 (0.001, 0.11)
% of Total Effect mediated	0.17%	2.92%	1.56%

Definition of abbreviations: AO, airflow obstruction; BD, bronchodilator; CI, confidence interval; NO_2_, nitrogen dioxide. ^1^ NO_2_ exposure included as a categorical variable by definition (≤5.8, >5.8 ppb). ^2^ Adjustment included body mass index, smoking status, pack-year group, sex, highest education, sampling weights, blood collection time and delivery, season and batch.

## Abbreviations

AO	Airflow obstruction
BD	Bronchodilator
CI	Confidence interval
COPD	Chronic obstructive pulmonary disease
CRP	C-reactive protein
CVD	Cardiovascular disease
FEV_1_	Forced expiratory volume in one second
FVC	Forced vital capacity
GM	Geometric mean
h	hours
IL	Interleukin
IQR	Interquartile range
ppb	Parts per billion
NO_2_	Nitrogen dioxide
PM_2.5_	Particulate matter less than 2.5 µm in diameter
RPM	Revolutions per minute
TAHS	Tasmanian longitudinal health study
TNF-α	Tumor necrosis factor-α
TRAP	Traffic-related air pollution
WHO	World Health Organization
